# Evidence for a multipotent mammary progenitor with pregnancy-specific activity

**DOI:** 10.1186/bcr3459

**Published:** 2013-08-15

**Authors:** Alice S Kaanta, Carl Virtanen, Laura M Selfors, Joan S Brugge, Benjamin G Neel

**Affiliations:** 1 Department of Medicine, Cancer Biology Program, Division of Hematology-Oncology, Beth Israel Deaconess Medical Center, Harvard Medical School, 330 Brookline Avenue, Boston, MA 02215, USA; 2 Department of Cell Biology, Harvard Medical School, 240 Longwood Avenue, Boston, MA 02115, USA; 3 Campbell Family Cancer Research Institute, Ontario Cancer Institute, Princess Margaret Cancer Center, University Health Network, 610 University Avenue, Toronto ON M5G 2M9, Canada; 4 Department of Medical Biophysics, University of Toronto, Ontario Cancer Institute, Princess Margaret Hospital, 610 University Avenue, Toronto, Ontario M5G 2M9, Canada

**Keywords:** Mammary progenitors, Mammary stem cells, Pregnancy, MMTVrtTA, H2BGFP

## Abstract

**Introduction:**

The mouse mammary gland provides a powerful model system for studying processes involved in epithelial tissue development. Although markers that enrich for mammary stem cells and progenitors have been identified, our understanding of the mammary developmental hierarchy remains incomplete.

**Methods:**

We used the MMTV promoter linked to the reverse tetracycline transactivator to induce H2BGFP expression in the mouse mammary gland. Mammary epithelial cells (MECs) from virgin mice were sorted by flow cytometry for expression of the mammary stem cell/progenitor markers CD24 and CD29, and H2BGFP. Sorted populations were analyzed for *in vivo* repopulation ability, expression of mammary lineage markers, and differential gene expression.

**Results:**

The reconstituting activity of CD24^+^/CD29^+^ cells in cleared fat pad transplantation assays was not distinguished in GFP^+^ compared to GFP^-^ subpopulations. However, within the CD24^+^/CD29^lo^ luminal progenitor-enriched population, H2BGFP^+^, but not H2BGFP^-^, MECs formed mammary structures in transplantation assays; moreover, this activity was dramatically enhanced in pregnant recipients. These outgrowths contained luminal and myoepithelial mammary lineages and produced milk, but lacked the capacity for serial transplantation. Transcriptional microarray analysis revealed that H2BGFP^+^/CD24^+^/CD29^lo^ MECs are distinct from H2BGFP^-^/CD24^+^/CD29^lo^ MECs and enriched for gene expression signatures with both the stem cell (CD24^+^/CD29^+^) and luminal progenitor (CD24^+^/CD29^lo^/CD61^+^) compartments.

**Conclusions:**

We have identified a population of MECs containing pregnancy-activated multipotent progenitors that are present in the virgin mammary gland and contribute to the expansion of the mammary gland during pregnancy.

## Introduction

During the lifetime of a female mammal, the mammary gland undergoes three distinct modes of growth [1]. In puberty, ductal elongation and branching establish the overall mammary architecture. After the female has reached sexual maturity, the mammary gland undergoes cyclic expansion and regression during the estrus/menstrual cycle. Pregnancy features dramatic proliferation and alveolar differentiation, which are critical for lactation. These processes are governed by different signals and hormones, and could involve the activity of different cell types [2,3].

The ability of the mammary gland to undergo repeated rounds of proliferation and remodeling in response to pregnancy was long cited as evidence for the existence of mammary stem cells. The Visvader and Eaves laboratories reported that subpopulations of mammary epithelial cells (MECs) within the CD24^+^/CD29^+^ or CD24^+^/CD49f^+^ compartments are capable of forming a fully functional mammary gland that can be serially transplanted [4,5]. These groundbreaking studies were followed by the discovery of additional markers for the purification and characterization of several MEC subtypes [6-9].

Whereas a mammary epithelial stem cell can be defined by its ability to establish a fully functional mammary tree *in vivo*, the identification and characterization of mammary progenitors (which are defined by their ability to replicate and differentiate, but not self-renew) require different types of assays. Lineage-restricted progenitors that differentiate into myoepithelial or luminal MECs have been identified, principally using *in vitro* assays [4-7]. The CD24^+^/CD29^lo^ population can be subdivided into populations of luminal progenitors and differentiated luminal MECs, depending on the expression or absence of CD61 (β3 integrin), respectively [6]. Luminal epithelial cells with different *in vitro* proliferative potential can be distinguished based on CD49b (α2 integrin) expression; CD24^+^/CD49b^+^, but not CD24^+^/CD49b^lo^, MECs form colonies on NIH 3T3 feeder cells [7]. CD14 and c-Kit expression have been used to identify prospective alveolar progenitors [8,9]. Although it was reported initially that CD24^+^/CD29^lo^ and CD24^+^/CD49f^lo^ MECs are unable to form outgrowths *in vivo,* more recent studies have demonstrated that these MECs can form small, branched mammary structures when co-injected with Matrigel into mammary fat pads [10,11]. Other groups transplanted mixed populations and inferred that the mammary gland contains progenitors that can give rise to mammary structures of different sizes and morphological characteristics [12,13].

Another approach to defining the *in vivo* activity of mammary stem cells and progenitors is to track MEC populations by lineage tracing. Van Keymeulen *et al.* recently conducted extensive studies of transgenic mice that inducibly express fluorescent proteins driven by promoters for known mammary lineage markers, including CK14, CK8 and CK18 [14]. This study provided evidence for the presence in the adult mammary gland of long-lived, unipotent luminal and myoepithelial progenitors that could contribute significantly to mammary gland expansion during puberty and pregnancy.

Adult stem cells also have been identified using pulse-chase assays that are based on the ability of stem cells to retain molecular labels significantly longer than bulk cells in tissues. Such ‘label retention’ is attributed either to slower cycling of stem cells or to asymmetric distribution of parental and daughter chromatids to stem cells and their progenitor progeny (the immortal strand hypothesis). Until recently, most label retention studies had been performed using DNA-based labels such as bromodeoxyuridine (BrdU) or tritiated thymidine. Previous reports that used such methods suggested the existence of label-retaining cells in the mammary gland that contribute to pregnancy, undergo mitosis and express steroid hormone receptors [15-17].

The development of transgenic mice expressing histone 2b fused to eGFP (H2BGFP) has permitted the isolation of live label-retaining cells, including epidermal and hematopoietic stem cells, by fluorescence-activated cell sorting (FACS) [18-20]. Using an analogous approach in an attempt to identify and purify mammary stem cells, we crossed H2BGFP transgenic mice with a strain expressing the reverse tetracycline transactivator under the control of the mouse mammary tumor virus promoter (MMTVrtTA). We chose this MMTVrtTA line because it reportedly expresses *tet* element-controlled transgenes in all MECs [21]. We found no evidence that label retention enriches for mammary stem cell activity, although the length of the ‘chase’ in our experiment might have been insufficient to isolate a mammary population of interest.

Nevertheless, in control experiments analyzing pubertal MMTVrtTA/H2BGFP mice, we made the unexpected discovery that H2BGFP was expressed primarily within the CD24^+^/CD29^+^ and CD24^+^/CD29^lo^ compartments, which contain mammary stem cells and progenitors, respectively. This unusual expression pattern led us to test whether the H2BGFP^+^ and H2BGFP^-^ MEC populations had distinct properties. Using mammary fat pad transplantation assays, we found that H2BGFP^+^/CD24^+^/CD29^lo^ cells contain a population of multipotent progenitors that give rise to mammary structures capable of differentiation and lactation, but which cannot be serially transplanted. The ability of these cells to produce such structures increases 5- to 10-fold if recipient mice are made pregnant. Therefore, our data suggest that the mammary glands of virgin pubertal mice contain a population of pregnancy-activated multipotent mammary progenitor cells.

## Methods

### Mice

MMTVrtTA mice [21] on FVB background were obtained from L. Chodosh (Abramson Cancer Institute). H2BGFP mice [18] in a CD-1 background were provided by E. Fuchs (Rockefeller University) and backcrossed onto FVB for a minimum of five generations prior to crossing with MMTVrtTA mice. To induce transgene expression, breeders and experimental mice were maintained on 2 mg/ml doxycycline (Research Products International Corp., Mount Prospect, IL, USA; 50213285). Mouse colonies were maintained according to federal, state, and institutional regulations and ethical approval for this study was obtained by the institutional review boards of Beth Israel Deaconess Medical Center and Harvard Medical School, respectively.

### Genotyping

Tail biopsies were obtained from transgenic mice, and dissolved in 0.5 ml tail lysis buffer (10 mM Tris + 100 mM NaCl + 10 mM EDTA + 0.5% SDS + 1 mg/ml proteinase K) at 60°C for >4 hours. After proteinase K treatment, 250 μl of 6 M NaCl were added to each sample, which was then inverted two to three times and incubated on ice for 10 minutes. Samples were centrifuged at 10,000 rpm in a microfuge for 10 minutes, and supernatants were added to 650 μl of isopropanol, inverted two to three times, and then centrifuged at top speed in a microfuge for 10 minutes. Pellets were air-dried and dissolved in 100 μl H_2_O. For H2BGFP genotyping, primers were: GFP-F: 5′-GCAAGGGCGAGGAGCTGTTCACC-3′, GFP-R: 5′-GGCGAGCTGCACGCTGCCGTCCTC-3′, expected band size 500 base pair (bp). For *tet* genotyping, primers were: t*TA*-F: 5′-CGCTGTGGGGCATTTTACTTTAG-3′, *tTA*-R: 5′-CATgTCCAgATCgAAATCgTC-3′, expected band size 450 bp. Control genotyping PCR (T-cell receptor delta) primers were: TCRD-F: 5′-CAAATGTTGCTTGTCTGGTG-3′, TCRD-R: 5′-gTCAgTCgAgTgCACAgTTT-3′, expected band size 200 bp. All genotyping PCRs were carried out as follows: 95°C 5 min, 95°C 45 sec, 58°C 45 sec, 72°C 60 sec, repeat from second 95°C step 25X, 72°C 5 min, 4°C final. PCR products were resolved on 2% agarose gels.

### Mammary gland harvesting and preparation

Mice were sacrificed and mammary glands were harvested. Lymph nodes were removed from inguinal gland number 4, mammary tissue was manually minced, placed in culture medium (DMEM/F12 + 500 ng/ml hydrocortisone + 10 ng/ml EGF + 10 μg/ml insulin + 20 ng/ml cholera toxin + 1% Pen/Strep) [4] with 600 U/ml collagenase and 200 U/ml hyaluronidase, and shaken at 37°C for 1 to 1.5 hrs. Cells from digested tissue were recovered by centrifugation and resuspended in a mixture of 0.25% Trypsin-EDTA (Gibco number 15050-065; Invitrogen, Carlsbad, CA, USA), 5 mg/ml dispase (Roche 04942078001; Roche, Basel, Switzerland) and 0.1 mg/ml DNAse (Sigma number D5319; Sigma-Aldrich, St Louis, MO, USA) for 5 to 7 minutes. Reactions were quenched with DMEM/F12 + 10% FBS, and cells were recovered by centrifugation, resuspended in Tris-buffered 0.64% NH_3_Cl for 3 minutes on ice, and then passed sequentially through 100 um and 40 um filters.

### Flow cytometry

Single cell suspensions were blocked in FACS buffer (PBS + 2 mM EDTA + 0.1% BSA) containing 1:25 normal rat serum (eBioscience, Inc., San Diego, CA, USA; 24-5555) and 1:25 anti-mouse FC receptor block (eBioscience 16-0161) for 10 minutes, and stained in FACS buffer containing directly conjugated antibodies for 20 minutes. Cells were resuspended in FACS buffer (containing 1 μg/ml DAPI for viability analysis) and analyzed on a BD Aria II or FACS Canto II using BD Aria software (BD Biosciences, San Jose, CA, USA). Antibodies included: CD24•PE (eBioscience 12-0242), CD29•APC (Biolegend, San Diego, CA, USA; 102215), CD49f•Biotin (eBioscience 13-0495), CD31•PeCy7 (eBioscience; 25-0311), CD45•PeCy7 (eBioscience 25-0451), Ter119•PeCy7 (eBioscience 25-5921) and Streptavidin APC-eFluor 780 (eBioscience 47-4317-82). Antibodies were used at the dilutions recommended by their manufacturer. Single cells were analyzed after gating out dead (DAPI^+^), hematopoietic (CD45^+^, Ter119^+^), and endothelial (CD31^+^) cells.

Antibodies tested in Figure S1 in Additional file 1: AlCam•PE (eBioscience 12-1661-81), amphiregulin (R&D Systems, Minneapolis, MN, USA; BAF989), CD14 (eBioscience 13-0141-80), CD24 (eBioscience 13-0242-80), CD44 (eBioscience 13-0441-81), CD49b (eBioscience; 13-5971-80), CD49f (eBioscience 13-0495), CD55 (eBioscience 12-0559-71), CD61 (eBioscience 13-0611-80), CD86 (eBioscience 13-0862-80), CD138/Syndecan•PE (R&D FAB2966P), CD164 (R&D BAF3118), c-Kit (eBioscience 13-1171-81), CXCL16 (R&D BAF503), E-cadherin (eBioscience 13-3249-80), EpCAM (eBioscience 13-5791-80), FGFR2•PE (R&D FAB684A), IL1R1•PE (R&D FAB7712P), IL33 (R&D BAF3626), Jag1•PE (eBioscience 12-3391-80), Muc1 (AbCam, Cambridge, UK; ab13970), Osteoactivin (R&D BAF2330), PRLR (R&D BAF1445), and Sca-1 (eBioscience). Secondary antibodies included: Streptavidin APC-eFluor 780 (eBioscience 47-4317-82), anti-rabbit Alexa750 (Invitrogen A-21039).

### Mammary fat pad transplants

Sorted cells were counted and resuspended in growth factor-reduced Matrigel (BD Biosciences, Franklin Lakes, NJ, USA; 354230). Three-week-old female mice between 10 to 12 g were obtained from Charles River Laboratories, Wilmington, MA, USA. Mice were anesthetized with 4 mg/10 g body weight Avertin (Sigma-Aldrich; T48402), and abdomens were shaved and cleaned with 70% ethanol and Betadine. An inverted Y-shaped incision was made along the thoracic-inguinal region, and the mammary glands were exposed. The nipple and mammary artery connecting the number 4 and number 5 glands were cauterized, and the epithelium was removed from the inguinal (number 4) mammary glands. The removed epithelium was fixed and stained with Carmine Alum solution (2 g/l Carmine + 5 g/l aluminum potassium sulfate) to verify complete clearing. Cells were injected into cleared fat pad pads in 10 μl volumes, using a Hamilton syringe (Hamilton, Reno, NV, USA). Incisions were closed with wound clips. For pain control, two doses of 0.5 mg/kg body weight Buprenorphine (Webster Veterinary, Devens, MA, USA; 07-867-1196) were administered in the 24 hrs after surgery. Mammary fat pads were harvested six weeks after injection, unless pregnancy induction required an earlier harvest date. Harvested tissues were either whole-mounted and scored for mammary outgrowths or dissociated for reanalysis and/or serial transplants [22].

### Pregnancy induction

Adult males were housed with post-transplant females three to four weeks after surgery. Plugs were checked daily to verify timing of pregnancies. Females were sacrificed and mammary glands were harvested between days 14 to 20 of the pregnancy.

### Whole mounts

Mice were sacrificed, and the number 4 mammary glands were dissected, spread onto slides, air-dried for 5 minutes and fixed in methyl Carnoy (1:3:6 glacial acetic acid, chloroform, methanol) for 4 to 24 hrs. Tissues were subjected to sequential washes with 100%, 75%, 50%, and 25% EtOH, followed by distilled water, and then stained overnight in Carmine Alum solution. Mammary glands were dehydrated sequentially in 70% and 100% EtOH, subjected to two washes with xylenes, and then mounted in Permount (Thermo Fisher Scientific, Waltham, MA, USA; SP15-100). Whole mounts were scored and imaged under a dissecting microscope [22]. All mammary outgrowths observed by Carmine Alum stain were confirmed by tissue sectioning and immunostaining.

### Statistical analysis

Mammary repopulating unit MRU frequencies were calculated using the ELDA (extreme limiting dilution analysis) method (http://bioinf.wehi.edu.au/software/elda/) [23].

### RNA analysis

RNA was harvested from sorted cells using the Qiagen RNeasy Micro Kit (Qiagen, San Diego, CA, USA; 74004), amplified using the NuGen RNA amplification system, and analyzed for gene expression on Illumina Whole Genome Mouse chips (Illumina, Inc., San Diego, CA, USA; WG6 v2r2). Processed arrays were checked for overall quality using R (v2.10.0) with the Bioconductor [24] and LUMI packages [25]. Data were imported into GeneSpring (v12.0, Agilent Technologies, Santa Clara, CA, USA) and normalized with a quantile-based algorithm. Because experimental samples were collected on two separate dates, a further batch correction was applied using a parametric empirical Bayes framework [26]. Pre-filtering was performed to remove probes with no expression in any of the groups under consideration (at least 80% of samples having an expression value greater than the 20th percentile). Hierarchical cluster diagrams were built using a Pearson centered distance measure under average linkage rules. Differentially expressed genes in GFP^+^ and GFP^-^ CD24^+^/CD29^lo^ cells were identified on a re-normalized and filtered subset of the whole data set using unpaired Student’s *t* test with Benjamini and Hochberg false discovery rate (FDR) [27] multiple testing correction (q <0.05). Microarray data have been made publicly available at the Gene Expression Omnibus (http://www.ncbi.nlm.nih.gov/geo) under the series identifier GSE42055.

For analysis of mammary subpopulations defined in Vaillant *et al*. [28], pre-processed data were downloaded from the Gene Expression Omnibus (GSE19446, http://www.ncbi.nlm.nih.gov/geo/) and analyzed using R (v.2.13.1). Differentially expressed genes in the CD24^+^/CD29^+^, CD24^+^/CD29^lo^/CD61^+^ and CD24^+^/CD29^lo^/CD61^+^ subpopulations were identified using one-way analysis of variance, post hoc Tukey honestly significant differences tests and a *P* value threshold of 0.05. These results were merged with the differentially expressed genes identified by the GFP^+^ and GFP^-^ CD24^+^/CD29^lo^ arrays, using the manufacturers’ unique probe identifiers. A representative single probe per gene was selected by highest variance across the GFP^+^ and GFP^-^ CD24^+^/CD29^lo^ samples. Fold-enrichment was defined as the observed frequency divided by the expected frequency. Enrichment *P* values were calculated with the hypergeometric distribution implemented in the phyper function in R. The background population set was the 16,245 unique genes that were identified after pre-filtering the GFP^+^ and GFP^-^ CD24^+^/CD29^lo^ data.

### qPCR

RNA was harvested from sorted cells using the Qiagen RNeasy Micro Kit, amplified using the NuGen RNA amplification system, and labeled using SYBR Green Master Mix (Applied Biosystems, Foster City, CA, USA; 4309155). qPCR reactions were performed by using an Applied Biosystems 7900HT. Primers were: *Esr1*-F 5′-TGGGCGACATTCTTCTCAA-3′, *Esr1*-R 5′-TGGACCAGAGGTACATCCATT-3′, *Prlr*-F 5′-GGTTATAGCATGATGACCTGCAT-3′, *Prlr*-R 5′- CAGTTCTTCAGACTTGCCCTTC-3′, *Areg*-F 5′-GCGAATGCAGATACATCGAG-3′, *Areg*-R 5′- CCACACCGTTCACCAAAGTA-3′, *Fgfr2-*F 5′-GAGCGCTGCCATTCAAGT-3′, *Fgfr2*-R 5′- TTGCTGTTGTTACTGCTGTTCC-3′, *Muc1*-F 5′-CTGTTCACCACCACCATGAC-3′, *Muc1*-R 5′-CTTGGAAGGGCAAGAAAACC-3′, *Wnt5a-*F 5′- ACGCTTCGCTTGAATTCCT-3′, *Wnt5a*-R 5′- CCCGGGCTTAATATTCCAA-3′, *Il1R1*-F 5′-CGAACCGTGAACAACACAAA-3′, *Il1R1*-R 5′- AATCTCCAGCGACAGCAGAG-3′, *Cav1*-F 5′-ACGACGACGTGGTCAAGATT-3′, *Cav1*-R 5′-CACAGTGAAGGTGGTGAAGC-3′, *Robo1*-F 5′-CCACCCACCAGACAGGAG-3′, *Robo1*-R 5′-GTATGAGGTGGGGAAATTGG-3′.

### Immunostaining

For tissue sections, mammary glands were fixed in methyl Carnoy, or, if previously stained with Carmine Alum, first unmounted in xylenes. Tissues were paraffin-embedded and sectioned. For antigen retrieval, slides were deparaffinized in xylenes, and rehydrated in decreasing concentrations of EtOH, followed by boiling for 20 minutes in 10 mM sodium citrate. For frozen sections, freshly dissected tissues were mounted in OCT compound (Sakura Finetek USA, Inc., Torrance, CA, USA; 4583), flash-frozen in liquid nitrogen and sectioned.

For cytospins, slides and coverslips were fixed in 4% PFA and rinsed with PBS for staining. Three-dimensional Matrigel cultures were fixed and stained as described by Debnath *et al.*[29]. Primary antibody staining was performed at 4°C overnight; secondary antibody staining was performed at room temperature for 1 hr.

Primary antibodies included: cytokeratin 8/TROMA-1 (Developmental Studies Hybridoma Bank TROMA-I-c), cytokeratin 18 (Covance, Inc., Emeryville, CA, USA; SIG-3466), cytokeratin 14 (Lifespan Biosciences, Inc., Seattle, WA, USA; LS-C22637), p63 (Biolegend 619001), β1 integrin (BD Biosciences 550531), estrogen receptor (Santa Cruz Biotechnology, Santa Cruz, CA, USA; SC-542), progesterone receptor (DAKO, Glostrup, Denmark; A0098), glucocorticoid receptor (Santa Cruz SC-1004), milk (Nordic Immunological Laboratories, Tilburg, The Netherlands; RAM/MSP), amphiregulin (Santa Cruz sc-25436). AlexaFluor™ secondary antibodies were used for visualization and imaging.

## Results

### MMTVrtTA induces H2BGFP expression in the mammary stem cell and progenitor compartments

We expressed H2BGFP in the mouse mammary gland by crossing mice that carry a tetracycline-inducible H2BGFP fusion gene [30] to mice expressing the reverse tetracycline transactivator under the control of the mouse mammary tumor virus promoter (MMTVrtTA) [21] (Figure S2 in Additional file 2). To induce H2BGFP expression, breeders and pups were treated with doxycycline throughout breeding, pregnancy, lactation, and after weaning until sacrifice. Mammary glands were harvested from double transgenic (MMTVrtTA/H2BGFP) virgin females at four weeks post-partum and analyzed by flow cytometry and immunostaining.

**Figure 1:**
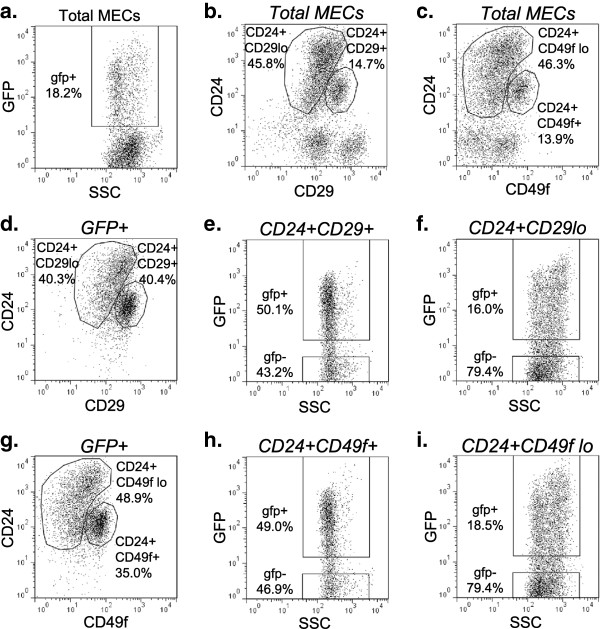
**Flow cytometric analysis of MMTVrtTA/H2BGFP expression within the mammary stem cell and progenitor populations.** Total mammary epithelial cell (MEC) population of four-week-old MMTVrtTA/H2BGFP mice, expressing: **(a)** H2BGFP **(b)** CD24/CD29 **(c)** CD24/CD49f. MMTVrtTA/H2BGFP expression within: **(e)** the CD24^+^/CD29^+^ gate; **(f)** the CD24^+^/CD29^lo+^ gate; **(h)** the CD24^+^/CD49f^+^ gate; and **(i)** the CD24^+^/CD49f^lo^ gate. Stem cell marker expression within the H2BGFP^+^ population: **(d)** CD24/CD29 **(g)** CD24/CD49f. H2BGFP, histone 2B-eGFP; MMTV, mouse mammary tumor virus promoter; rtTA, reverse tetracycline transactivator.

For flow cytometric analysis, mammary glands were dissociated to single cell suspensions and stained for CD24, CD29, and CD49f (Figure 1). These markers divided MECs into the subpopulations (Figure 1b, c) reported previously [4,5]. As expected, H2BGFP was not detected in mammary cells from single transgenic or uninduced double transgenic mice (data not shown). In contrast to a previous report that this MMTVrtTA transgene drives ubiquitous expression in MECs [21], H2BGFP expression was detected in only a fraction (18 ± 3.1%) of MECs from double transgenic mice (Figure 1a; also see Discussion).

The vast majority of H2BGFP^+^ MECs were found in either the mammary stem cell (CD24^+^/CD29^+^, CD24^+^/CD49f^+^) or progenitor (CD24^+^/CD29^lo^, CD24^+^/CD49f^lo^) compartments. Of the H2BGFP^+^ cells, 47 ± 11% were CD24^+^/CD29^+^, while 39 ± 11% were CD24^+^/CD29^lo^ (n = 10; representative flow cytometric profile is shown in Figure 1d). Conversely, 55 ± 6.7% of CD24^+^/CD29^+^ MECs and 15 ± 4.4% of CD24^+^/CD29^lo^ MECs were H2BGFP^+^, respectively (n = 10; representative flow cytometric profiles are shown in Figures 1e, f). Similarly, 36 ± 7.1% of the H2BGFP^+^ cells were CD24^+^/CD49f^+^ and 50 ± 1.7% were CD24^+^/CD49f^lo^ (n = 4; representative flow cytometry profile is shown in Figure 1g), while 46 ± 3.6% of CD24^+^/CD49f^+^ MECs and 21 ± 2.7% of CD24^+^/CD49f^lo^ MECs were H2BGFP^+^ (n = 4; representative flow cytometric profiles are shown in Figures 1h, i). Although each subpopulation exhibited a range of H2BGFP levels, the maximum fluorescence intensity was higher in the CD24^+^/CD29^lo^ and CD24^+^/CD49f^lo^ (progenitor-containing) populations than in the CD24^+^/CD29^+^ and CD24^+^/CD49f^+^ (stem cell-containing) compartments (Figure 1e, f, h, i).

**Figure 2:**
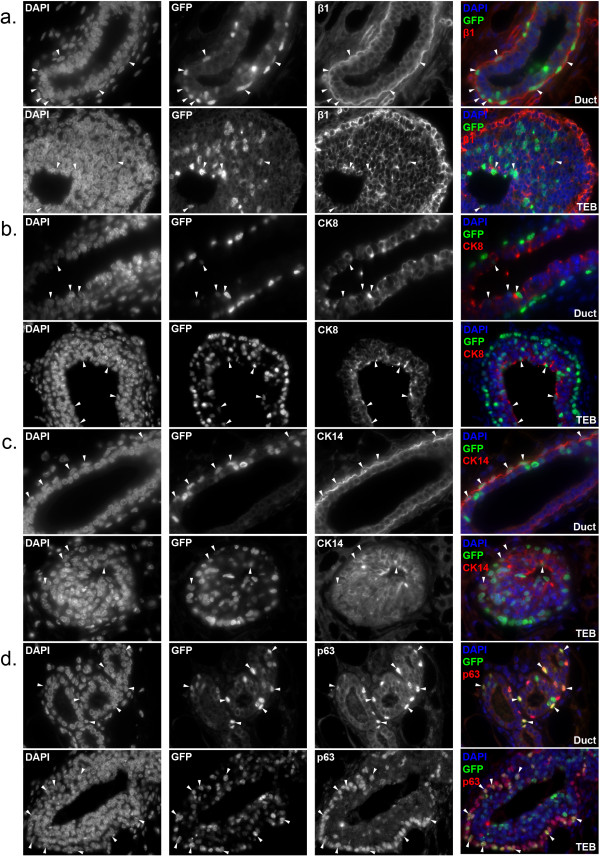
**Expression of lineage markers in mammary glands of MMTVrtTA/H2BGFP mice.** Mammary glands were harvested from four-week-old double transgenic females treated with doxycycline, sectioned and immunostained for: **(a)** β1 integrin **(b)** CK8 **(c)** CK14 or **(d)** p63. Each pair of micrographs depicts ducts (upper) and terminal end buds (lower), at 60× magnification. Note that, as expected, cytoplasmic (CK8, CK14) and cell surface (β1 integrin) lineage markers do not co-localize with H2BGFP in cells that co-express both proteins; only with p63, which is a nuclear protein, does co-expression result in co-localization with H2BGFP. In all panels, representative co-expressing cells are indicated by arrowheads. H2BGFP, histone 2B-eGFP; MMTV, mouse mammary tumor virus promoter; rtTA, reverse tetracycline transactivator.

To further characterize H2BGFP^+^ MECs, we also analyzed the pattern of H2GFP expression *in situ*. Mammary glands from four-week-old, doxycycline-induced, MMTVrtTA/H2BGFP females were sectioned and immunostained for β1 integrin (CD29), CK8, CK14, and p63. Consistent with the flow cytometry data, H2BGFP was expressed mosaically and at a range of levels, and the majority of H2BGFP^+^ cells co-expressed β1 integrin (Figure 2a). H2BGFP^+^ MECs were detected in the basal layer of the ductal tree and in the cap cells of the terminal end buds (TEB), as well as in the luminal layer. Moreover, the luminal (CK8^+^; Figure 2b), myoepithelial (CK14^+^; Figure 2c), and basal/cap cell (p63^+^; Figure 2d) populations each contained H2BGFP + MECs (arrowheads). These data are consistent with our finding that H2BGFP^+^ cells fall predominantly within the CD24^+^/CD29^+^ and CD24^+^/CD29^lo^ compartments.

**Figure 3:**
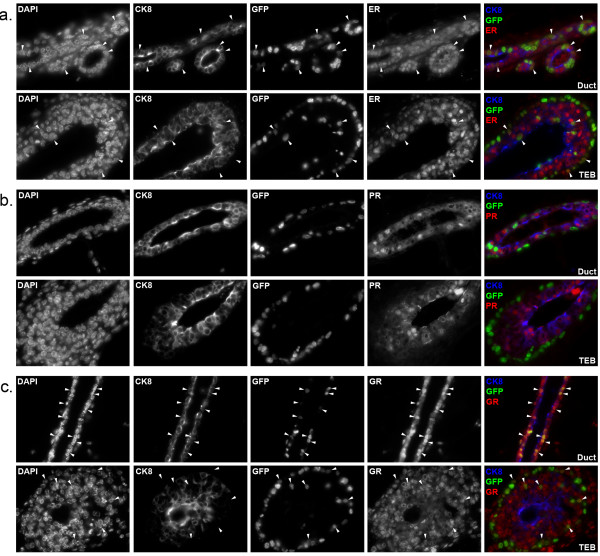
**Expression of steroid hormone receptors in the mammary glands of MMTVrtTA/H2BGFP mice.** Mammary glands were harvested from four-week-old double transgenic females treated with doxycycline, sectioned and immunostained for: **(a)** estrogen receptor (ER) **(b)** progesterone receptor (PR) and **(c)** glucocorticoid receptor (GR). Micrographs depict ducts (left) and terminal end buds (right) at 60× magnification. Arrowheads indicate representative co-expressing cells. H2BGFP, histone 2B-eGFP; MMTV, mouse mammary tumor virus promoter; rtTA, reverse tetracycline transactivator.

We also examined how H2GFP expression correlated with the expression of steroid hormone receptors that are associated with differentiated MEC populations and play important roles in mammary biology [4,5,31]. Mammary glands from four-week-old double transgenic mice were sectioned and immunostained for CK8 to mark the luminal compartment, along with estrogen receptor (ER) (Figure 3a), progesterone receptor (PR) (Figure 3b) or glucocorticoid receptor (GR) (Figure 3c), which activates the MMTV promoter and is important for mammary proliferation during pregnancy [32]. In these pubertal mice, most cells in the ducts and TEBs were ER^+^ and GR^+^, and H2BGFP was co-expressed with ER and GR in some of these cells (Figure 3a, c, arrowheads). By contrast, H2BGFP^+^ cells consistently failed to express PR (Figure 3b).

### MMTVrtTA/H2BGFP expression identifies MECs with differential repopulation activity

The mammary stem cell/progenitor-containing compartments identified by CD24 and CD29 expression are mixed populations. For example, the CD24^+^/CD29^+^ compartment has a MRU frequency of 1/64 [4], and also contains myoepithelial progenitors [14]. Differential CD61 expression divides the CD24+/CD29^lo^ compartment into luminal progenitors (CD24^+^/CD29^lo^/CD61^+^) and mature luminal cells (CD24^+^/CD29^lo^/CD61^-^), as demonstrated by *in vitro* assays [6]. Because MMTVrtTA/H2BGFP expression also subdivided these populations, we asked whether mammary stem cells or progenitors could be enriched further based on H2BGFP. To this end, we tested the repopulating activity of all four H2BGFP populations (H2BGFP^+^/CD24^+^/CD29^+^, H2BGFP^-^/CD24^+^/CD29^+^, H2BGFP^+^/CD24^+^/CD29^lo^ and H2BGFP^-^/CD24^+^/CD29^lo^) by injecting limiting dilutions of FACS-purified cells into the cleared mammary fat pads of three-week-old mice. Recipients were maintained on doxycycline to continue transgene induction.

Fat pads that had received transplants were harvested six weeks post-transplantation, stained with Carmine Alum to visualize outgrowths and scored for size (Table 1, Figure S3 in Additional file 3, Table S1 in Additional file 4). Because some outgrowths were quite small, recipient fat pads also were sectioned and immunostained for mammary epithelial markers to verify their mammary origin and to visualize the structure of all outgrowths. The MRU frequency of each population was calculated, using the ELDA method [23,33,34]; similar results were obtained by assuming single-hit Poisson statistics (data not shown). In order to increase the size and visibility of the mammary glands, pregnancy was induced in some recipients, three to four weeks after transplantation. To determine whether pregnancy affected repopulation, data from virgin and pregnant recipients were analyzed separately.

**Table 1:** Limiting dilution transplants of MMTVrtTA/H2BGFP populations

**Population**	**Number of cells**	**Number of glands**	**MRU frequency (confidence interval)**
*Virgin recipients*			
**a. H2GFP**^**+**^**/CD24**^**+**^**/CD29**^**+**^	10	3/16	1/105 (70 - 160)
	20	5/12	
	50	3/12	
	100	6/10	
	200	9/12	
**b. H2GFP**^**-**^**/CD24**^**+**^**/CD29**^**+**^	10	2/12	1/80 (60 - 130)
	20	2/14	
	50	5/12	
	100	9/12	
	200	9/10	
**c. H2GFP**^**+**^**/CD24**^**+**^**/CD29**^**lo**^	10	0/14	1/1,600 (500 - 4,900)
	20	0/12	
	50	0/12	
	100	1/12	
	200	2/14	
**d. H2GFP**^**-**^**/CD24**^**+**^**/CD29**^**lo**^	10	0/14	None
	20	0/12	
	50	0/12	
	100	0/14	
	200	0/14	
*+ Pregnancy*			
**e. H2GFP**^**+**^**/CD24**^**+**^**/CD29**^**+**^	10	1/4	1/60 (35 - 100)
	20	3/6	
	50	5/8	
	100	5/8	
	200	6/6	
**f. H2GFP**^**-**^**/CD24**^**+**^**/CD29**^**+**^	10	3/8	1/140 (80 - 240)
	20	3/6	
	50	2/8	
	100	4/8	
	200	4/8	
**g. H2GFP**^**+**^**/CD24**^**+**^**/CD29**^**lo**^	10	1/6	1/350 (160 -790)
	20	0/8	
	50	1/6	
	100	1/8	
	200	3/6	
**h. H2GFP**^**-**^**/CD24**^**+**^**/CD29**^**lo**^	10	0/6	None
	20	0/8	
	50	0/6	
	100	0/6	
	200	0/6	
*Virgin Recipients vs. Pregnancy - 2*^*nd*^*Experimental Set*			
**i. H2GFP**^**+**^**/CD24**^**+**^**/CD29**^**lo**^	10	0/14	1/3,800 (500 - 27,000)
*Virgin*	20	0/12	
	50	1/12	
	100	0/12	
	200	0/8	
**j. H2GFP**^**+**^**/CD24**^**+**^**/CD29**^**lo**^	10	2/12	1/280 (170 - 480)
*+Pregnancy*	20	2/14	
	50	4/14	
	100	4/14	
	200	4/14	

Mammary repopulating ability of H2BGFP^+^/CD24^+^/CD29^+^ (a, e), H2BGFP^-^/CD24^+^/CD29^+^ (b, f), H2BGFP^+^/CD24^+^/CD29^lo^ (c, g, i) and H2BGFP^-^/CD24^+^/CD29^lo^ (d, h, j) populations in virgin mice (a-d, i) and pregnant mice (e-h, j). H2BGFP, histone 2B-eGFP; MMTV, mouse mammary tumor virus promoter; MRU, mammary reproducing unit; rtTA, reverse tetracycline transactivator.

We found that the repopulation abilities of H2BGFP^+^/CD24^+^/CD29^+^ and H2BGFP^-^/CD24^+^/CD29^+^ populations were similar in virgin and pregnant recipients (Table 1a, b, e, f) and comparable to established repopulation frequencies for (unfractionated) CD24^+^/CD29^+^ cells [4]. Based on these data, we conclude that MMTVrtTA/H2BGFP expression in the CD24^+^/CD29^+^ compartment does not label MECs with different repopulating activity.

**Figure 4:**
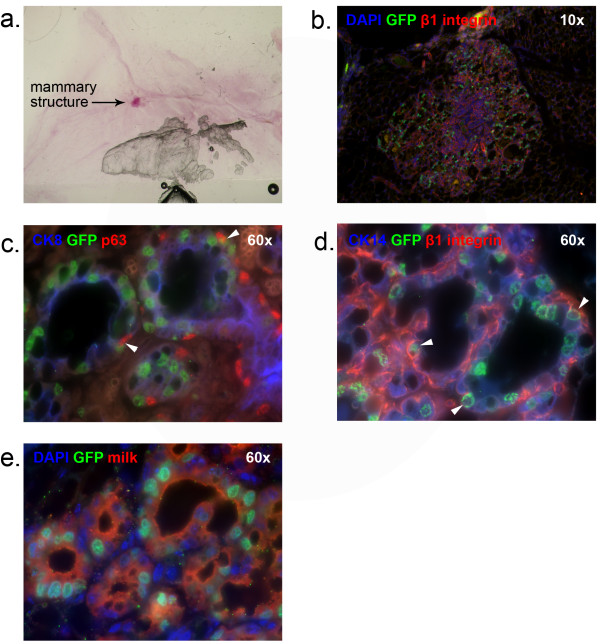
***In vivo *outgrowth of H2BGFP**^**+**^**/CD24**^**+**^**/CD29 **^**lo **^**cells.** Outgrowths from H2BGFP^+^/CD24^+^/CD29^lo^ cells were stained with Carmine Alum for whole-mount analysis, then sectioned and immunostained to analyze their epithelial origin and composition. **(a)** mammary fat pad whole mount **(b)** DAPI, GFP and β1 integrin staining **(c)** CK8, GFP and p63 staining **(d)** CK14, GFP and β1 integrin staining **(e)** DAPI, GFP and milk staining. Panel b, 10× magnification. Panels c-e, 60× magnification. Arrowheads indicate representative co-expressing cells. DAPI, 4',6-diamidino-2-phenylindole; GFP, green fluorescent protein; H2BGFP, histone 2B-eGFP.

To determine whether MMTVrtTA/H2BGFP expression could identify previously unidentified mammary progenitors with *in vivo* activity, we also tested the H2BGFP^+^ and H2BGFP^-^ CD24^+^/CD29^lo^ populations in transplantation experiments. Although CD24^+^/CD29^lo^ MECS were initially believed to be incapable of *in vivo* mammary gland reconstitution [4], other studies have since discovered that this population can develop mammary outgrowths upon transplantation, under certain conditions [10,11] (see Discussion). We discovered that, in contrast to CD24^+^/CD29^+^ cells, the ability of H2BGFP^+^/CD24^+^/CD29^lo^ MECs to give rise to multilineage outgrowths was nearly 5-fold higher in pregnant (1/350), compared with virgin (1/1,600), mice (Table 1c, g). Outgrowths from H2BGFP^+^/CD24^+^/CD29^lo^ MECs were much smaller than glands derived from CD24^+^/CD29^+^ cells (Figure S3 in Additional file 3, Table S1 in Additional file 4), but contained all mammary lineages and were able to produce milk (Figure 4).

### H2BGFP^+^/CD24^+^/CD29^lo^ MECs give rise to mammary outgrowths preferentially in pregnant mice

To directly address whether H2BGFP^+^/CD24^+^/CD29^lo^ MECs expand selectively during pregnancy, we performed an additional set of transplantation experiments (n = 3), focusing specifically on the H2BGFP^+^/CD24^+^/CD29^lo^ population. Half of the mice injected with H2BGFP^+^/CD24^+^/CD29^lo^ MECs were made pregnant post-transplant to allow direct comparison of H2BGFP^+^/CD24^+^/CD29^lo^ cell repopulating activity in virgin vs. pregnant mice (Table 1, bottom). In agreement with the first set of experiments, the MRU frequency of H2BGFP^+^/CD24^+^/CD29^lo^ MECs was 1/280 in pregnant mice, while in virgin recipients, H2BGFP^+^/CD24^+^/CD29^lo^ MECs produced only a single outgrowth (Table 1i, j). We conclude that MMTVrtTA-driven expression of H2BGFP in the CD24^+^/CD29^lo^ compartment labels a population containing multipotent stem cells or progenitors that expand preferentially during pregnancy.

### H2GFP^+^/CD24^+^/CD29^lo^ cells do not self-renew

Stem cells, unlike progenitors, have the ability to self-renew. In the hematopoietic system, there is a hierarchy of stem cells with long-term, intermediate-term and short-term reconstitution ability, respectively [35]. To test whether MMTVrtTA-driven expression of H2GFP might mark MECs with differential self-renewal ability, we compared the serial transplantation ability of the three cell populations (H2BGFP^+^/CD24^+^/CD29^+^, H2BGFP^-^/CD24^+^/CD29^+^, and H2BGFP^+^/CD24^+^/CD29^lo^) capable of forming mammary outgrowths. Each MEC population was isolated by FACS from four-week-old MMTVrtTA/H2BGFP females and injected in 500-cell aliquots into 10 cleared fat pads of syngeneic mice. Recipients were treated with doxycycline to promote H2BGFP expression. After six weeks, transplanted fat pads were harvested and pooled according to transplanted cell type, analyzed by flow cytometry, and re-transplanted. MMTVrtTA/H2BGFP populations were serially transplanted twice in two experiments, and tertiary transplants were performed in one experiment.

**Figure 5:**
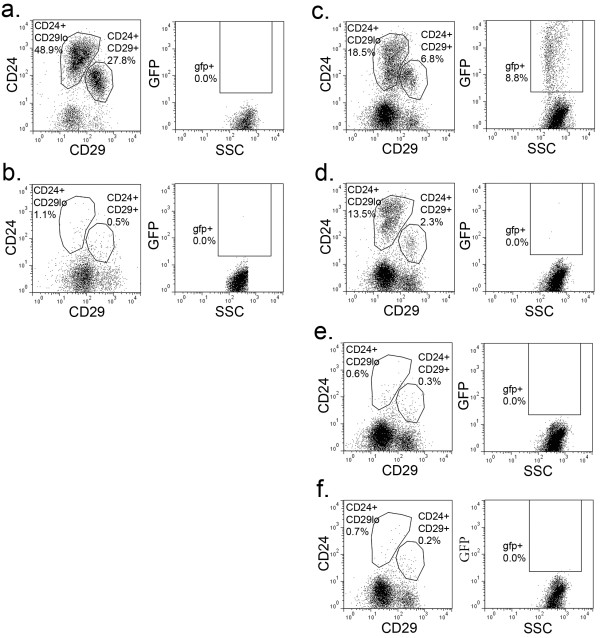
**Serial transplants of H2BGFP/CD24/CD29 populations.** Flow cytometric analysis of CD24/CD29 (left) and H2BGFP expression (right) in: **(a)** wild type control mammary glands; **(b)** cleared mammary fat pads; and recipients of: **(c)** H2BGFP^+^/CD24^+^/CD29^+^ cells; **(d)** H2BGFP^-^/CD24^+^/CD29^+^ cells; **(e)** H2BGFP^+^/CD24^+^/CD29^lo^ cells; or **(f)** H2BGFP^-^/CD24^+^/CD29^lo^ cells. H2BGFP, histone 2B-eGFP.

The H2BGFP^+^/CD24^+^/CD29^+^ and H2BGFP^-^/CD24^+^/CD29^+^ populations each gave rise to serially transplantable mammary glands (Figure 5). Surprisingly, only H2BGFP^+^/CD24^+^/CD29^+^ cells gave rise to mammary glands that expressed H2BGFP. To eliminate the possibility that the apparent H2BGFP^-^/CD24^+^/CD29^+^ outgrowths were the product of endogenous MECs from incompletely cleared mammary fat pads, genomic DNA was harvested from mammary glands after tertiary transplants and tested by PCR for the MMTVrtTA and H2BGFP transgenes. Both transgenes were present in H2BGFP^+^/CD24^+^/CD29^+^- and H2BGFP^-^/CD24^+^/CD29^+^-derived glands, demonstrating their transgenic origin (data not shown). These results suggest that H2BGFP expression was repressed permanently in the H2BGFP^-^/CD24^+^/CD29^+^ population. We conclude that both CD24^+^/CD29^+^ populations are capable of long-term stem cell activity, and H2BGFP expression within this compartment does not, at least at this level of analysis, distinguish subpopulations of CD24^+^/CD29^+^ cells with differential biological activity.

By contrast, the H2BGFP^+^/CD24^+^/CD29^lo^ population was unable to serially transplant, indicating that the multilineage outgrowths that arise from this compartment derive from a multipotent progenitor, not a stem cell.

### Gene expression analysis is consistent with biologically distinct roles for H2BGFP^+^/CD24^+^/CD29^lo^ and H2BGFP^-^/CD24^+^/CD29^lo^ populations

**Figure 6:**
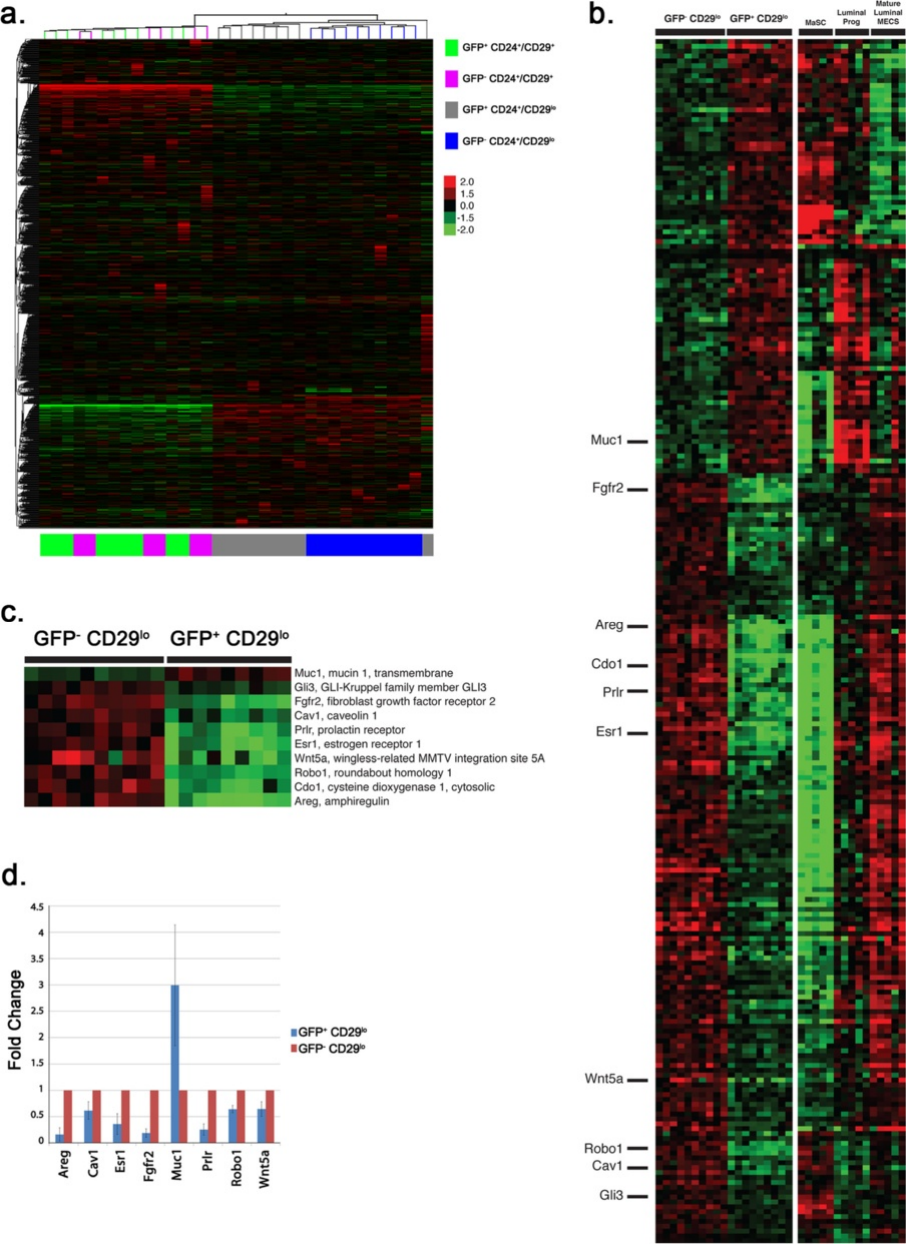
**Gene expression differences segregate MMTVrtTA/H2BGFP MECs into distinct subpopulations. (a)** Gene expression data from the four MMTVrtTA/H2BGFP populations were analyzed using an unsupervised two-way hierarchical clustering of 35,927 filtered probes on the Illumina Mouse Whole Genome (WG-6 version 2 release 2) microarray. Samples in the CD29^+^CD24^-^ populations can be seen to segregate into two distinct subpopulations based on the H2BGFP marker, whereas H2BGFP does not affect the clustering of CD29^+^CD24^+^ samples. **(b**-**d)** Differentially expressed genes between the H2BGFP^+^/CD24^+^/CD29^lo^ and H2BGFP^-^/CD24^+^/CD29^lo^ populations were compared with those found in a publicly available data set [36] consisting of isolated mammary stem cell-enriched (MaSC), luminal progenitor (lum prog) and mature luminal mammary epithelial cell (lum mature MEC) populations. The resulting 247 genes in common were plotted as a heat map **(b)**. Ten genes **(c)** were chosen for further validation and follow-up analyses based on their association with mammary gland development and differentiation. **(d)** Validation of eight of these genes by quantitative RT-PCR. H2BGFP, histone 2B-eGFP; MMTV, mouse mammary tumor virus promoter; rtTA, reverse tetracycline transactivator.

The above data provide evidence that H2BGFP^+^ CD24^+^/CD29^lo^ MECs contain a subpopulation of pregnancy-activated multipotent progenitors. To characterize these cells further, RNA was isolated from the four MEC subpopulations of four-week-old mice, and transcriptional profiling was performed using the Illumina Mouse Microarray platform. Analysis of the resulting data by unsupervised hierarchical clustering segregated the samples into subpopulations defined by CD24, CD29 and H2BGFP expression (Figure 6a). Because we observed no functional differences between the H2GFP^+^ and H2GFP^-^ CD24^+^/CD29^+^ populations, their failure to form a separate cluster by unsupervised analysis was not surprising, and suggests that H2BGFP expression in the CD24^+^/CD29^+^ population is a stochastic phenomenon. By contrast, H2BGFP^+^/CD24^+^/CD29^lo^ and H2BGFP^-^/CD24^+^/CD29^lo^ MECs segregated into distinct clusters, consistent with their different behavior in transplantation experiments.

We focused our subsequent analyses on the gene expression differences between GFP^+^ and GFP^-^ CD24^+^/CD29^lo^ cells, respectively, and found that 654 probes (representing 587 unique transcripts) had significantly (FDR-adjusted *P* <0.05) different expression levels in these two populations (Additional file 5). Of these probes, 150 (representing 131 unique transcripts) had a greater than 2-fold difference. When cross-referenced with the Gene Ontology database, ten of the differentially expressed genes were found to be associated with mammary gland development and differentiation. Each of these transcripts, except for *Mucin* 1 *(Muc1*), was expressed at much lower levels in H2BGFP^+^/CD24^+^/CD29^lo^ cells (Figure 6c and Additional file 6). These included such well-documented mediators of mammary gland development as the *Estrogen receptor 1* (*Esr1*), *Fibroblast growth factor receptor 2* (*Fgfr2*), *Prolactin receptor* (*Prlr*), and *Amphiregulin* (*Areg*) genes. To validate these observations, mRNA from H2BGFP^+^/CD24^+^/CD29^lo^ and H2BGFP^-^/CD24^+^/CD29^lo^ MECs was analyzed by qPCR (Figure 6d). These assays confirmed the microarray data for eight of the ten mammary developmental genes: *Areg*, *Caveolin 1* (*Cav1*), *Esr1*, *Fgfr2*, *Mucin 1* (*Muc1*), *Prlr*, *Roundabout homolog 1* (*Robo1*) and *Wingless-type MMTV integration site family, member 5A* (*Wnt5a*). In addition, MMTVrtTA/H2BGFP MECs from each of the four populations were cytospun onto slides and immunostained for proteins whose transcripts were expressed differentially in the H2BGFP^+^/CD24^+^/CD29^lo^ and H2BGFP^-^/CD24^+^/CD29^lo^ populations and for mammary lineage markers (Figure S4 in Additional file 7). Again, higher levels of *Areg* and *Esr1* were found in H2BGFP^-^/CD24^+^/CD29^lo^ MECs than in H2BGFP^+^/CD24^+^/CD29^lo^ MECs.

We next compared the gene expression profiles of the H2GFP^+^/CD24^+^/CD29^lo^ cells and three mammary cell subpopulations defined by Shackleton *et al.*[4] and Vaillant *et al.*[28], and characterized by Lim *et al.*[36]: mammary stem-cell enriched (MaSC) (CD24^+^/CD29^e^/CD61^+^), luminal progenitor (CD24^+^/CD29^lo^/CD61^+^), and mature luminal (CD24^+^/CD29^+^/CD61^-^) cells (Figure 6b). Differentially expressed genes in H2BGFP^+^/CD24^+^/CD29^lo^ and H2BGFP^-^/CD24^+^/CD29^lo^ MECs were enriched for genes that distinguish the CD24^+^/CD29^+^, CD24^+^/CD29^lo^/CD61^+^ and CD24^+^/CD29^e^/CD61^+^ subpopulations (*P* = 1.07e^-06^). Transcripts expressed at higher levels in H2BGFP^-^/CD24^+^/CD29^lo^ cells (compared with H2GFP^-^/CD24^e^/CD29^lo^ cells) were 2.8-fold enriched for genes that were expressed highly in CD24^+^/CD29^lo^/CD61^+^ MECs, at lower levels in CD24^+^/CD29^lo^/CD61^-^ mature cells and at lowest levels in the CD24^+^/CD29^+^ stem cell compartment (*P* = 9.91e^-08^). The transcripts expressed at higher levels in H2BGFP^+^/CD24^+^/CD29^lo^ MECs also were 2.8-fold enriched for genes expressed highly in the stem cell compartment, at lower levels in the CD24^e^/CD29^lo^/CD61^+^ population, and at lowest levels in the mature luminal cells (*P* = 8.75e^-06^).

Although these data indicate that H2BGFP^+^/CD24^+^/CD29^lo^ cells are more similar to CD24^+^/CD29^lo^/CD61^+^ MECs than to CD24^+^/CD29^lo^/CD61^-^ MECs, most notably in their lower levels of transcripts for mammary developmental genes such as *Areg*, *Esr1*, *Fgfr2* and *Prl*, H2BGFP expression in the CD24^+^/CD29^lo^ population did not correlate with CD61 expression (Figure S1 in Additional file 1). Therefore, H2BGFP^+^/CD24^+^/CD29^lo^ cells share transcriptional characteristics with mammary stem cells and luminal-restricted progenitors, a phenotype that might be predicted for a multipotent progenitor, but one clearly distinct from the CD24^+^/CD29^+^ and CD24^+^/CD29^lo^/CD61^+^ populations.

## Discussion

We have identified and characterized a MEC population with properties of a pregnancy-activated multipotent progenitor. By studying mammary development in MMTVrtTA/H2BGFP transgenic mice, we discovered a subset of CD24^+^/CD29^lo^ cells possessing the unique ability to develop small, multilineage mammary outgrowths, ranging in size from a small alveolar cluster to a half-grown mammary gland, that can produce milk, yet have no self-renewal activity. The ability of these cells to form glandular outgrowths increases 5- to 10-fold when recipient mice are made pregnant. Transcriptional profiling provides additional evidence that the H2BGFP^+^ and H2GFP^-^ CD24^+^/CD29^lo^ populations are biologically distinct, with H2GFP^+^ cells expressing lower levels of key transcripts involved in mammary differentiation and higher levels of transcripts found in the CD24^+^/CD29^+^, mammary stem cell-enriched, compartment.

These H2BGFP^+^/CD24^+^/CD29^lo^ MECs are identifiable in mice in early puberty, which suggests that they arise during or before pubertal development and remain dormant until pregnancy. The ability of these progenitors to give rise to mammary outgrowths three to four weeks after they are transplanted also is consistent with an ability to remain dormant until activated by pregnancy stimuli.

Our observation that MMTVrtTA is active in mammary stem cells and pregnancy-activated progenitors conflicts with a previous report that the same strain of MMTVrtTA is expressed homogeneously in all MECs [21]. In that study, the MMTVrtTA transgene-induced expression of tetracycline-driven LacZ, and an enzymatic assay was used to detect transgene expression. The enzymatic LacZ assay is probably more sensitive than flow cytometric detection of H2BGFP. Moreover, if the β-galactosidase reaction was not in the linear range in the earlier experiments, it might not have differentiated between levels of protein expression, resulting in the erroneous conclusion that MMTVrtTA transgene activity was uniform in the mammary gland. In any event, the interpretation of studies that assumed that this MMTVrtTA strain directs transgene expression ‘uniformly’ in the mammary gland should be reconsidered in light of our data that expression is largely restricted to stem cells and progenitors.

The molecular basis for selective MMTV-driven labeling of stem cells and pregnancy-activated progenitors remains to be elucidated. In separate experiments, wherein the H2BGFP strain was crossed to other rtTA transgenic mice, H2BGFP expression was found in significantly fewer MECs and did not correlate with specific mammary lineages (data not shown). Therefore, the pattern of H2BGFP expression observed in our study appears to be determined by the MMTVrtTA transgene.

The MMTV promoter contains PR and GR binding sites, and mammary development during pregnancy is regulated by progesterone and glucocorticoids, suggesting a possible mechanism behind MMTV activity in the mouse mammary gland; however, we found no correlation between H2BGFP expression and PR or GR expression. Alternatively, the insertion site of this particular MMTVrtTA transgene could underlie the expression pattern observed in this study.

Some investigators have hypothesized that mammary stem cells, rather than progenitors, are activated during pregnancy for alveoli production [37,38]. Others, however, suggest that the establishment of the mammary architecture of pregnancy utilizes a process distinct from the proliferation of puberty and estrus, involving the activity of different cell types [39,40]. Wagner *et al.* suggested the existence of pregnancy-activated progenitors by using WAP-Cre transgenic mice in combination with a Rosa26-lox-lacZ allele to tag MECs in pregnant glands [40]. After an initial pregnancy to activate the WAP-Cre transgene, lineage tracing was used to identify a population of pregnancy-initiated MECs (PI-MECs) that could give rise to alveoli in successive pregnancies. These cells also could form fully functional mammary glands and were found subsequently to be CD24^+^/CD49f^+^[41,42], leading these authors to conclude that PI-MECs represent a mixed population of alveoli-forming progenitors and stem cells. Our H2BGFP^+^/CD24^+^/CD29^-^ cells, by contrast, do not give rise to serially transplantable mammary outgrowths, demonstrating that this population lacks mammary stem cells. Because we did not trace the dynamics of H2BGFP^+^/CD24^+^/CD29^lo^ MECs, we cannot determine the fate of these progenitor-derived structures following parturition.

While this work was in progress, Vaillant *et al.* reported that CD24^+^/CD29^-^ cells can form limited mammary structures *in vivo* only when co-injected with Matrigel, while the repopulating ability of CD24^+^/CD29^+^ cells is the same with or without Matrigel co-injection [10]. Jeselsohn *et al.* also found that CD24^+^/CD49f^lo^ MECs developed mammary outgrowths only in the presence of Matrigel [11], whereas Spike *et al.* reported that Matrigel could increase the mammary repopulation activity content of embryonic mammary cells [43]. These results suggest that the mammary gland contains progenitors that require additional signals or growth factors to survive or proliferate, and could explain why the ability of CD24^+^/CD29^lo^ cells to form outgrowths was missed in earlier studies [4]. Although these studies demonstrated that CD24^+^/CD29^lo^ MECs can form mammary outgrowths following transplant into virgin females, they did not assess the effects of pregnancy on the rate (or detection) of repopulation.

Our data show that this Matrigel-dependent outgrowth-generating ability is restricted to a subpopulation within the CD24^+^/CD29^lo^ compartment. Furthermore, the proliferation and development of outgrowths from these cells is increased by pregnancy. It is possible that Matrigel is required for the engraftment of H2BGFP^+^/CD24^+^/CD29^lo^ cells *in vivo*, but based on our data, we conclude that pregnancy is still the critical trigger for development of mammary structures able to pass the threshold of detection in most outgrowths.

The existence of mammary progenitors with hormone- or environment-dependent activity is supported by a recent and extensive lineage-tracing study, which concluded that mammary stem cells derive from an embryonic CK14^+^ lineage, whereas CK8 cells only give rise to luminal progenitors [14]. Cells from the CK8^+^ lineage also can contribute to alveoli in successive pregnancies, but are unable to form mammary structures *in vivo* without co-injection of other MECs. In another study, Sleeman *et al.* showed that CD24^+^ MECs co-transplanted with Matrigel can give rise to mammary outgrowths with a wide range of sizes, suggesting that mammary stem cells/progenitors with different *in vivo* growth potentials exist within the CD24^+^ compartment [13]. These findings, in conjunction with our data, suggest that the mammary gland contains progenitors that undergo proliferation only when presented with specific stimuli, such as pregnancy hormones or paracrine signals from other MECs. Although Matrigel clearly can supply some signals required for mammary progenitor survival or proliferation, in our study, pregnancy was the primary driver of mammary outgrowths from H2BGFP^+^/CD24^+^/CD29^lo^ cells.

The functional differences in MEC subpopulations identifiable by MMTVrtTA/H2BGFP expression were verified by gene expression analysis. By unsupervised hierarchical clustering, samples from the H2BGFP^+^ and H2GFP^-^ CD24^+^/CD29^lo^ populations segregated independently, consistent with their functional differences in transplantation experiments. Significantly, genes with critical roles in mammary development, including *Areg*, *Esr1* and *Prlr*, were expressed at lower levels in H2BGFP^+^/CD24^+^/CD29^e^ cells, which suggests that these cells are less differentiated than H2BGFP^-^/CD24^+^/CD29^lo^ MECs. H2BGFP^+^/CD24^+^/CD29^lo^ cells also shared partial gene signatures with the CD24^+^/CD29^+^ (stem cell) and CD24^+^/CD29^lo^/CD61^+^ (luminal progenitor) populations. In particular, genes expressed at lower levels in the H2BGFP^+^/CD24^+^/CD29^lo^ cells were expressed at low levels in CD24^+^/CD29^+^ (stem cell-enriched) MECs, whereas genes expressed at higher levels in H2BGFP^+^/CD24^+^/CD29^lo^ cells were expressed at higher levels in CD24^+^/CD29^lo^/CD61^+^ MECs. Because H2BGFP expression in the CD24^+^/CD29^lo^ population did not correlate with CD61 expression (Figure S1 in Additional file 1), these results suggest that H2BGFP^+^/CD24^+^/CD29^lo^ cells have an intermediate phenotype between mammary stem cells and luminal progenitors, as expected for a multipotent progenitor.

We attempted to identify a marker that could separate the H2BGFP^+^ and H2BGFP^-^ populations in the CD24^+^/CD29^lo^ compartment by FACS, without use of our transgenic model. To this end, we tested antibodies against antigens found by the microarray (CD14, AREG, AlCAM, CD55, CXCL16, CD164, CD138, PRLR) or that have been identified as potential mammary lineage markers (CD49b, CD24, Sca-1, c-kit). However, no marker proved suitable for isolation of the H2BGFP^+^/CD24^+^/CD29^lo^ population (Figure S1 in Additional file 1). The only notable result was that all H2BGFP^+^/CD24^+^/CD29^lo^ MECs, as well as a significant fraction of H2BGFP^-^/CD24^+^/CD29^lo^ MECs, were found to be CD14^+^. Other studies have found that CD14^+^ MECs demonstrate an alveolar phenotype *in vitro*, but the *in vivo* role of these cells is currently unknown [8,9].

While this manuscript was in review, Dos Santos and colleagues used an H2BGFP label retention approach to isolate an enriched mammary stem cell population employing the K5 promoter to induce H2BGFP expression rather than the MMTV promoter. The CD24^+^/CD29^+^ subpopulation that was isolated using label retention was found to have a mammary repopulation frequency of 1/33, as compared to the 1/70 described by Shackleton *et al.*[4]. Transcriptional profiling of these cells led to the identification of additional cell surface markers CD1d, CD22, and CD59a that could further purify mammary stem cells [20]. In our studies, we were unable to find CD24^+^/CD29^+^ MECs with enhanced mammary repopulation abilities, through either MMTVrtTA/H2BGFP expression or MMTVrtTA/H2BGFP label retention, though it is possible our ‘chase’ was insufficient in the label retention studies.

It has been hypothesized that the mammary stem cells responsible for establishing the mammary architecture during puberty are directly responsible for all subsequent mammary proliferation [37,38]. However, our data suggest that the developing mammary gland contains multipotent pregnancy-activated progenitors that give rise to milk-producing mammary structures *in vivo*. We conclude that the mammary stem cell hierarchy contains progenitors not only for different MEC lineages, but also for the specific functions and structures involved in lactation. These progenitors are of particular interest in light of the evidence that early pregnancy can protect from breast cancer, presumably from a reduction in the proliferative potential of stem cells and/or the likelihood that progenitors that are potential targets for malignant transformation. MECs that proliferate actively in response to pregnancy might be prone to oncogenesis, and therefore merit further investigation on both scientific and medical grounds.

## Conclusions

We have provided evidence for a multipotent mammary progenitor present in the glands of pre-pubertal virgin females, which, when transplanted into the mammary fat pad, gives rise to milk-producing mammary structures containing multiple cell lineages at a dramatically higher frequency during pregnancy. This progenitor population is distinguished from previously identified mammary lineages by *in vivo* function and gene expression analysis, and might represent a target cell in mammary tumorigenesis. Further study is required to better elucidate this progenitor’s role in normal mammary development and oncogenesis.

## Abbreviations

bp: Base pair; BSA: Bovine serum albumin; CI: Confidence interval; DAPI: 4',6-diamidino-2-phenylindole; DMEM: Dulbecco’s modified Eagle’s medium; EGF: Epidermal growth factor; ER: Estrogen receptor; FACS: Fluorescence-activated cell sorting; FBS: Fetal bovine serum; FDR: False discovery rate; GFP: Green flourescent protein; GR: Glucocorticoid receptor; H2BGFP: Histone 2B enhanced GFP; MaSC: Mammary stem cell; MEC: Mammary epithelial cell; MMTV: Mouse mammary tumor virus promoter; MRU: Mammary repopulating unit; PBS: Phosphate-buffered saline; PR: Progesterone receptor; rtTA: Reverse tetracycline transactivator; TEB: Terminal end bud.

## Competing interests

The authors declare that they have no competing interests.

## Authors’ contributions

ASK conducted the experiments, analyzed data, and drafted the manuscript. LMS and CV performed the bioinformatic/statistical analysis of the microarray data. BGN conceived the study, and BGN and JSB participated in its design, coordination, and data analysis and helped to draft and edit the manuscript. All authors read and approved the final manuscript.

## Supplementary Material

Additional file 1: Figure S1 Candidate markers for separating H2BGFP^+^/CD24^+^/CD29^lo^ and H2BGFP^-^/CD24^+^/CD29^lo^ populations. Mammary epithelial cells (MECs) from 4-week-old MMTVrtTA/H2BGFP females were isolated, stained with a panel of antibodies against surface proteins, and analyzed by flow cytometry for their ability to distinguish the H2BGFP^+^/CD24^+^/CD29^lo^ (green) and H2BGFP^-^/CD24^+^/CD29^lo^ (red) populations. Markers included previously identified stem cell/progenitor markers and differentially expressed genes identified from microarray analyses (see Figure 6). H2BGFP, histone 2B-eGFP; MMTV, mouse mammary tumor virus promoter; rtTA, reverse tetracycline transactivator. Click here for file

Additional file 2: Figure S2 Schematic of MMTVrtTA/H2BGFP mice. The MMTVrtTA strain (tet-on) was crossed with tet-responsive H2BGFP transgenic mice, resulting in tetracycline/doxycycline-activated, MMTV-driven expression of H2BGFP. H2BGFP, histone 2B-eGFP; MMTV, mouse mammary tumor virus promoter; rtTA, reverse tetracycline transactivator. Click here for file

Additional file 3: Figure S3 Mammary gland scoring system. Mammary outgrowths were harvested 6 weeks post-transplant, fixed and stained with Carmine Alum. Mammary glands were scored for outgrowth size based on the percentage of the mammary fat pad filled with epithelium, as indicated. Click here for file

Additional file 4: Table S1 Mammary outgrowth sizes in limiting dilution transplants. Size of mammary outgrowths from H2BGFP^+^/CD24^+^/CD29^+^**(a**, **e)**, H2BGFP^-^/CD24^+^/CD29^+^**(b**, **f)**, H2BGFP^+^/CD24^+^/CD29^lo^**(c**, **g**, **i)** and H2BGFP^-^/CD24^+^/CD29^lo^**(d**, **h**, **j)** populations in virgin mice **(a**-**d**, **i)** and pregnant mice **(e**-**h**, **j)**. H2BGFP, histone 2B-eGFP. Click here for file

Additional file 5 Hierarchical clustering of probes found to be significantly differentially expressed between the CD29 + CD24- GFP + versus CD29 + CD24- GFP- populations. Click here for file

Additional file 6 **Differentially expressed genes between the H2BGFP**^**+**^**/CD24**^**+**^**/CD29**^**lo **^**and H2BGFP-/CD24+/CD29**^**lo **^**populations compared with those found in a publicly available data set consisting of isolated mammary stem cell-enriched (MaSC), luminal progenitor (lum prog) and mature luminal mammary epithelial cell (lum mature MEC) populations.** H2BGFP, histone 2B-eGFP. Click here for file

Additional file 7: Figure S4 Cytospins of MMTVrtTA/H2BGFP populations. MMTVrtTA/H2BGFP mammary epithelial cell (MEC) populations were isolated by fluorescence-activated cell sorting (FACS), cytospun onto slides, fixed and immunostained for mammary lineage markers and microarray hits. The percentage of cells from each subpopulation that stain positive for each marker is indicated. H2BGFP, histone 2B-eGFP; MMTV, mouse mammary tumor virus promoter; rtTA, reverse tetracycline transactivator. Click here for file
