# Supplementary figures and images for: Evidence for a multipotent mammary progenitor with pregnancy-specific activity
Source: Breast Cancer Res. 2013 Aug 15;15(4):R65. doi: 10.1186/bcr3459 (PMC3979108; doi:10.1186/bcr3459)

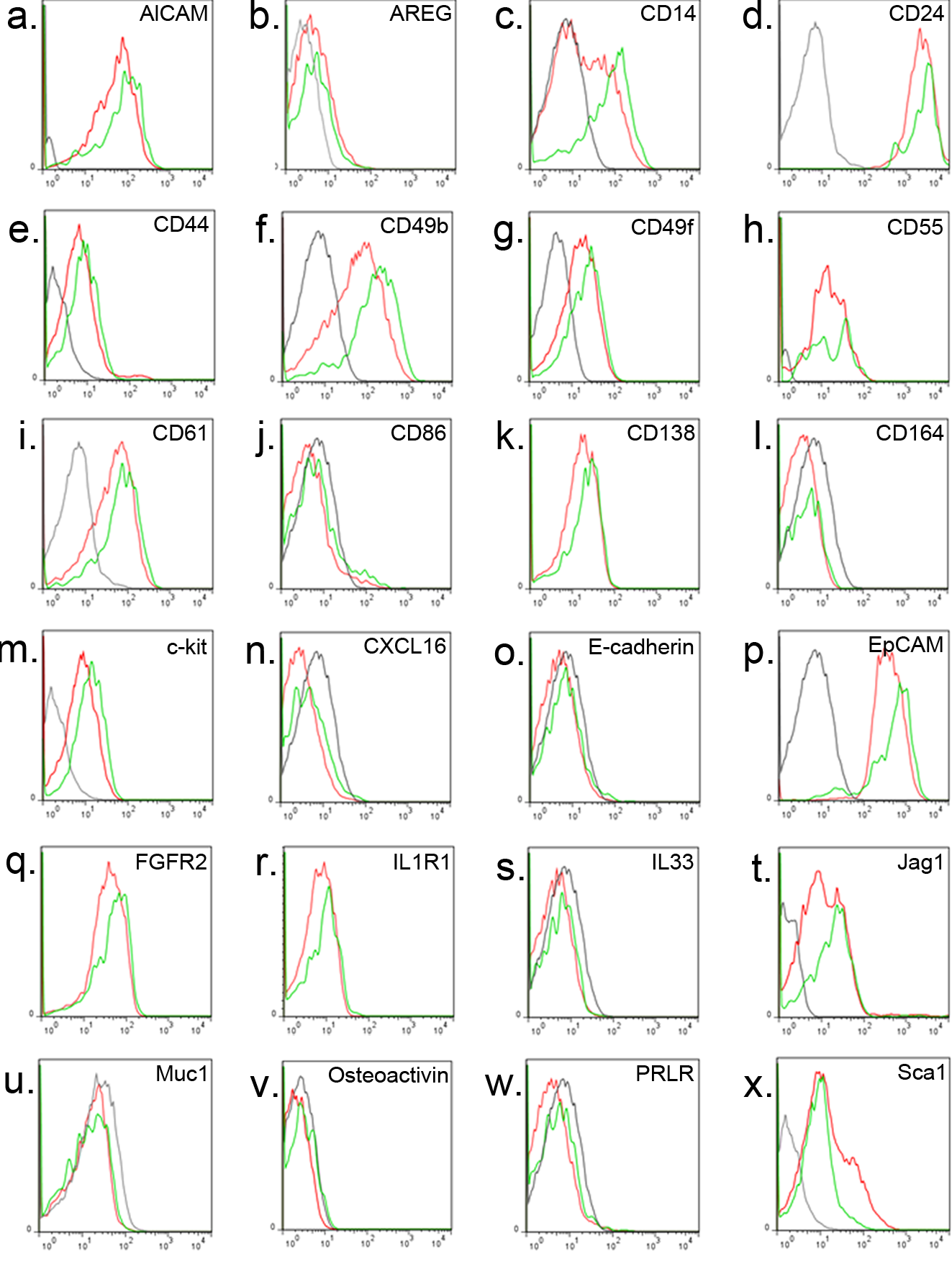

Supplement: Additional file 1: Figure S1 — Candidate markers for separating H2BGFP+/CD24+/CD29lo and H2BGFP-/CD24+/CD29lo populations. Mammary epithelial cells (MECs) from 4-week-old MMTVrtTA/H2BGFP females were isolated, stained with a panel of antibodies against surface proteins, and analyzed by flow cytometry for their ability to distinguish the H2BGFP+/CD24+/CD29lo (green) and H2BGFP-/CD24+/CD29lo (red) populations. Markers included previously identified stem cell/progenitor markers and differentially expressed genes identified from microarray analyses (see Figure 6). H2BGFP, histone 2B-eGFP; MMTV, mouse mammary tumor virus promoter; rtTA, reverse tetracycline transactivator. [file bcr3459-S1.png]

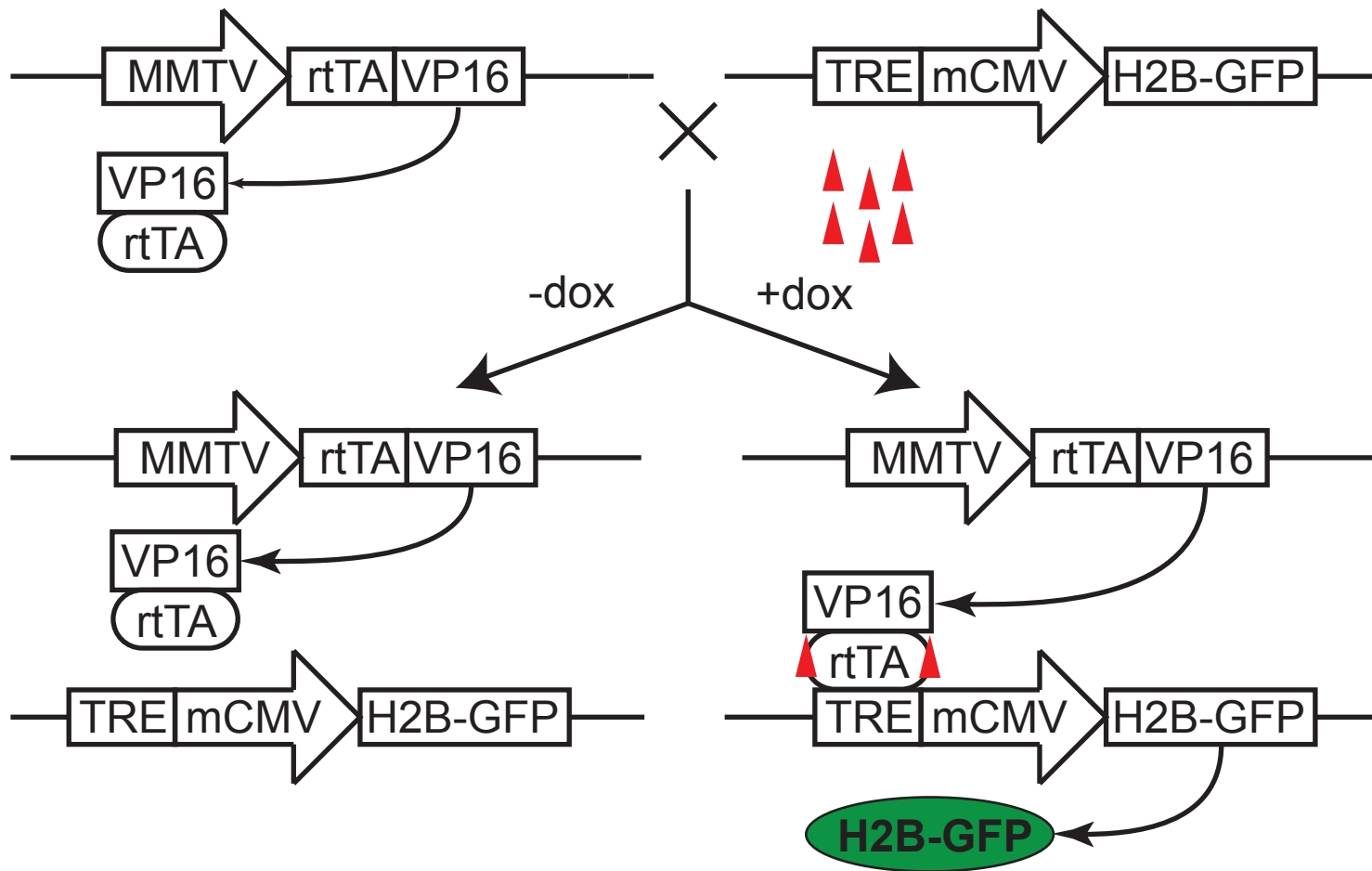

Supplement: Additional file 2: Figure S2 — Schematic of MMTVrtTA/H2BGFP mice. The MMTVrtTA strain (tet-on) was crossed with tet-responsive H2BGFP transgenic mice, resulting in tetracycline/doxycycline-activated, MMTV-driven expression of H2BGFP. H2BGFP, histone 2B-eGFP; MMTV, mouse mammary tumor virus promoter; rtTA, reverse tetracycline transactivator. [file bcr3459-S2.pdf]

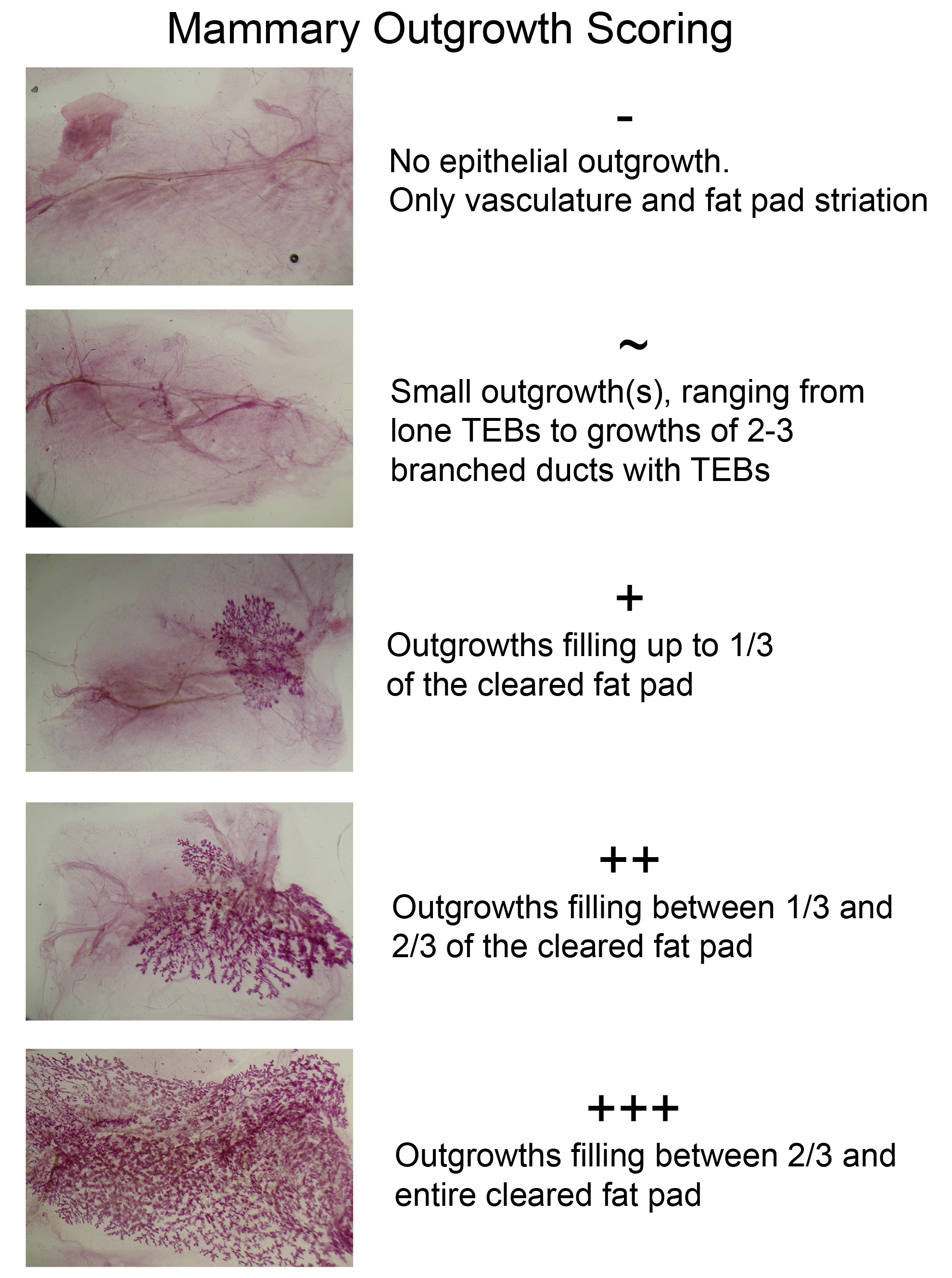

Supplement: Additional file 3: Figure S3 — Mammary gland scoring system. Mammary outgrowths were harvested 6 weeks post-transplant, fixed and stained with Carmine Alum. Mammary glands were scored for outgrowth size based on the percentage of the mammary fat pad filled with epithelium, as indicated. [file bcr3459-S3.png]

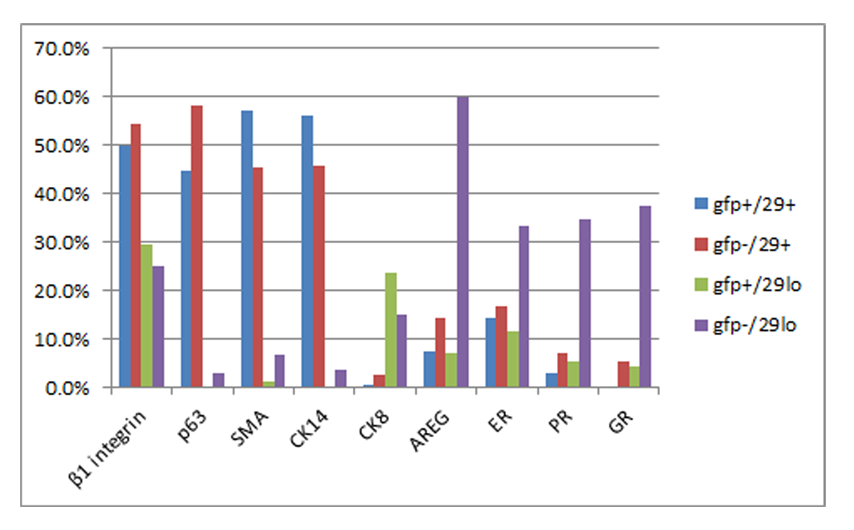

Supplement: Additional file 7: Figure S4 — Cytospins of MMTVrtTA/H2BGFP populations. MMTVrtTA/H2BGFP mammary epithelial cell (MEC) populations were isolated by fluorescence-activated cell sorting (FACS), cytospun onto slides, fixed and immunostained for mammary lineage markers and microarray hits. The percentage of cells from each subpopulation that stain positive for each marker is indicated. H2BGFP, histone 2B-eGFP; MMTV, mouse mammary tumor virus promoter; rtTA, reverse tetracycline transactivator. [file bcr3459-S7.png]
