# Supplementary material for: Evidence for a multipotent mammary progenitor with pregnancy-specific activity
Source: Breast Cancer Res. 2013 Aug 15;15(4):R65. doi: 10.1186/bcr3459 (PMC3979108; doi:10.1186/bcr3459)
Supplement: Additional file 4: Table S1 — Mammary outgrowth sizes in limiting dilution transplants. Size of mammary outgrowths from H2BGFP+/CD24+/CD29+(a, e), H2BGFP-/CD24+/CD29+(b, f), H2BGFP+/CD24+/CD29lo(c, g, i) and H2BGFP-/CD24+/CD29lo(d, h, j) populations in virgin mice (a-d, i) and pregnant mice (e-h, j). H2BGFP, histone 2B-eGFP. [file bcr3459-S4.doc]

Supplementary Table 1. Outgrowth Size Scoring of Transplants.

|  |  |  | **Outgrowth Size** | | | | |
| --- | --- | --- | --- | --- | --- | --- | --- |
| **Population** | **# cells** |  | **-** | **~** | **+** | **++** | **+++** |
| *Virgin Recipients* |  |  |  |  |  |  |  |
| **a. H2GFP^+^/CD24^+^/CD29^+^** | 10 |  | 13 | 1 | 2 | 0 | 0 |
|  | 20 |  | 7 | 1 | 2 | 1 | 1 |
|  | 50 |  | 9 | 1 | 1 | 0 | 1 |
|  | 100 |  | 4 | 0 | 0 | 4 | 2 |
|  | 200 |  | 3 | 2 | 4 | 2 | 1 |
|  |  |  |  |  |  |  |  |
| **b. H2GFP^-^/CD24+/CD29^+^** | 10 |  | 10 | 1 | 1 | 0 | 0 |
|  | 20 |  | 12 | 1 | 0 | 0 | 1 |
|  | 50 |  | 7 | 0 | 2 | 1 | 2 |
|  | 100 |  | 3 | 1 | 4 | 3 | 1 |
|  | 200 |  | 1 | 4 | 1 | 2 | 0 |
|  |  |  |  |  |  |  |  |
| **c. H2GFP^+^/CD24^+^/CD29^lo^** | 10 |  | 14 | 0 | 0 | 0 | 0 |
|  | 20 |  | 12 | 0 | 0 | 0 | 0 |
|  | 50 |  | 14 | 0 | 0 | 0 | 0 |
|  | 100 |  | 11 | 1 | 0 | 0 | 0 |
|  | 200 |  | 12 | 2 | 0 | 0 | 0 |
|  |  |  |  |  |  |  |  |
| **d. H2GFP^-^/CD24^+^/CD29^lo^** | 10 |  | 14 | 0 | 0 | 0 | 0 |
|  | 20 |  | 12 | 0 | 0 | 0 | 0 |
|  | 50 |  | 12 | 0 | 0 | 0 | 0 |
|  | 100 |  | 14 | 0 | 0 | 0 | 0 |
|  | 200 |  | 14 | 0 | 0 | 0 | 0 |
| *+ Pregnancy* |  |  |  |  |  |  |  |
| **e. H2GFP^+^/CD24^+^/CD29^+^** | 10 |  | 3 | 0 | 0 | 1 | 0 |
|  | 20 |  | 3 | 1 | 1 | 1 | 0 |
|  | 50 |  | 3 | 1 | 0 | 1 | 3 |
|  | 100 |  | 3 | 0 | 0 | 1 | 4 |
|  | 200 |  | 0 | 0 | 3 | 2 | 3 |
|  |  |  |  |  |  |  |  |
| **f. H2GFP^-^/CD24+/CD29^+^** | 10 |  | 5 | 0 | 2 | 1 | 0 |
|  | 20 |  | 5 | 2 | 0 | 0 | 1 |
|  | 50 |  | 6 | 0 | 0 | 1 | 1 |
|  | 100 |  | 4 | 0 | 1 | 2 | 1 |
|  | 200 |  | 4 | 0 | 1 | 0 | 3 |
|  |  |  |  |  |  |  |  |
| **g. H2GFP^+^/CD24^+^/CD29^lo^** | 10 |  | 5 | 0 | 0 | 1 | 0 |
|  | 20 |  | 8 | 0 | 0 | 0 | 0 |
|  | 50 |  | 5 | 1 | 0 | 0 | 0 |
|  | 100 |  | 7 | 1 | 0 | 0 | 0 |
|  | 200 |  | 3 | 1 | 2 | 0 | 0 |
|  |  |  |  |  |  |  |  |
| **h. H2GFP^-^/CD24^+^/CD29^lo^** | 10 |  | 6 | 0 | 0 | 0 | 0 |
|  | 20 |  | 8 | 0 | 0 | 0 | 0 |
|  | 50 |  | 6 | 0 | 0 | 0 | 0 |
|  | 100 |  | 6 | 0 | 0 | 0 | 0 |
|  | 200 |  | 6 | 0 | 0 | 0 | 0 |
|  |  |  |  |  |  |  |  |
| *Virgin Recipients vs. Pregnancy – 2^nd^ Experiment* | | | | | | | |
| **i. H2GFP^+^/CD24^+^/CD29^lo^** | 10 |  | 14 | 0 | 0 | 0 | 0 |
| *virgin* | 20 |  | 12 | 0 | 0 | 0 | 0 |
|  | 50 |  | 11 | 1 | 0 | 0 | 0 |
|  | 100 |  | 12 | 0 | 0 | 0 | 0 |
|  | 200 |  | 8 | 0 | 0 | 0 | 0 |
|  |  |  |  |  |  |  |  |
| **j. H2GFP^+^/CD24^+^/CD29^lo^** | 10 |  | 10 | 2 | 0 | 0 | 0 |
| *+pregnancy* | 20 |  | 12 | 1 | 0 | 0 | 1 |
|  | 50 |  | 11 | 4 | 0 | 0 | 0 |
|  | 100 |  | 10 | 1 | 1 | 2 | 0 |
|  | 200 |  | 9 | 1 | 3 | 0 | 1 |

**Supplementary Table 1. Mammary Outgrowth Sizes in Limiting Dilution Transplants.** Size of mammary outgrowths from H2BGFP^+^/CD24^+^/CD29^+^ **(a, e)**, H2BGFP^-^/CD24^+^/CD29^+^ **(b, f)**, H2BGFP^+^/CD24^+^/CD29^lo^ **(c, g, i)** and H2BGFP^-^/CD24^+^/CD29^lo^ **(d, h, j)** populations in virgin mice **(a-d, i)** and pregnant mice **(e-h, j)**
